# Fifth-week immunogenicity and safety of anti-SARS-CoV-2 BNT162b2 vaccine in patients with multiple myeloma and myeloproliferative malignancies on active treatment: preliminary data from a single institution

**DOI:** 10.1186/s13045-021-01090-6

**Published:** 2021-05-17

**Authors:** Fulvia Pimpinelli, Francesco Marchesi, Giulia Piaggio, Diana Giannarelli, Elena Papa, Paolo Falcucci, Martina Pontone, Simona Di Martino, Valentina Laquintana, Antonia La Malfa, Enea Gino Di Domenico, Ornella Di Bella, Gianluca Falzone, Fabrizio Ensoli, Branka Vujovic, Aldo Morrone, Gennaro Ciliberto, Andrea Mengarelli

**Affiliations:** 1 Microbiology and Virology Unit, Dermatological Clinical and Research Department, IRCCS San Gallicano Institute, Rome, Italy; 2 Hematology Unit, Department of Research and Clinical Oncology, IRCCS Regina Elena National Cancer Institute, Via Elio Chianesi 53, 00144 Rome, Italy; 3 SAFU Unit, Department of Research and Innovative Technologies, IRCCS Regina Elena National Cancer Institute, Rome, Italy; 4 Clinical Trial Center, Biostatistics and Bioinformatics Unit, Scientific Direction, IRCCS Regina Elena National Cancer Institute, Rome, Italy; 5 Biological Tissue and Liquid Bank, Scientific Direction, IRCCS Regina Elena National Cancer Institute, Rome, Italy; 6 Pharmacy Unit, Medical Direction, IRCCS Regina Elena National Cancer Institute and San Gallicano Institute, Rome, Italy; 7 Medical Direction, IRCCS Regina Elena National Cancer Institute and San Gallicano Institute, Rome, Italy; 8 Scientific Direction, IRCCS San Gallicano Institute, Rome, Italy; 9 Scientific Direction, IRCCS Regina Elena National Cancer Institute, Rome, Italy

**Keywords:** mRNA vaccine, COVID-19, Hematological malignancy

## Abstract

**Background:**

Safety and immunogenicity of BNT162b2 mRNA vaccine are unknown in hematological patients; both were evaluated prospectively in 42 patients with multiple myeloma (MM) and 50 with myeloproliferative malignancies (MPM) (20 chronic myeloid leukemias and 30 myeloproliferative neoplasms), all of them on active anti-cancer treatment, in comparison with 36 elderly controls not suffering from cancer. Subjects serologically and/or molecularly (by nasal/throat swab) positives at basal for SARS-CoV-2 were excluded. Primary endpoint was to compare titers of neutralizing anti-SARS-CoV-2 IgG and seroprotection rates among the cohorts at 3 and 5 weeks from first dose.

**Methods:**

Titration was done using LIAISON® SARS-CoV-2 S1/S2 IgG test, a quantitative chemiluminescent immunoassay approved by FDA on the basis of robust evidences of concordance (94.4%) between the test at cutoff of 15 AU/mL and the Plaque Reduction Neutralization Test 90% at 1:40 ratio. Cutoff of 15 AU/mL was assumed to discriminate responders to vaccination with a protective titer. Cohorts were compared using Fisher’ exact test and the Mann–Whitney test as appropriated. Geometric mean concentrations (GMCs), geometric mean ratios and response rates after 1st and 2nd dose were compared in each cohort by Wilcoxon and McNemar tests, respectively.

**Results:**

At 5 weeks, GMC of IgG in elderly controls was 353.3 AU/mL versus 106.7 in MM (*p* = 0.003) and 172.9 in MPM patients (*p* = 0.049). Seroprotection rate at cutoff of 15 AU/mL was 100% in controls compared to 78.6% in MM (*p* = 0.003) and 88% in MPM patients (*p* = 0.038). In terms of logarithm of IgG titer, in a generalized multivariate linear model, no gender effect was observed (*p* = 0.913), while there was a significant trend toward lower titers by increasing age (*p* < 0.001) and in disease cohorts with respect to controls (MM: *p* < 0.001 and MPM: *p* < 0.001). An ongoing treatment without daratumumab was associated with higher likelihood of response in MM patients (*p* = 0.003). No swabs resulted positive on each time point. No safety concerns were observed.

**Conclusions:**

BNT162b2 has demonstrated to be immunogenic at different extent among the cohorts. Response was 88% and robust in MPM patients. MM patients responded significantly less, particularly those on anti-CD38-based treatment. These latter patients should be advised to maintain masks and social distancing regardless of vaccination status, and their cohabiting family members need to be vaccinated in order to reduce the risk of contagion from the family. Additional boosters and titer monitoring could be considered.

*Trial registration* Study was formally approved by the IRCCS Central Ethical Committee of Regione Lazio in January 2021 (Prot. N-1463/21).

## Background

It is highly desirable that vaccines against SARS-CoV-2, hopefully including its variants, could work also in hematological malignancy patients. Such patients were not enrolled in the registration trials, and, so far, no data on COVID-19 vaccines for this vulnerable population have been published, except for chronic lymphocytic leukemia [1]. Therefore, recommendations and expectations in clinical practice are based on evidences coming from the immune response to COVID-19 infection and non-COVID-19 vaccines [2].

In a large retrospective multicenter Italian cohort study, overall COVID-19-attributable mortality rate in 536 patients with hematological malignancies was 37% [3]. Patients with progressive disease status and diagnosis of acute myeloid leukemia, lymphomas and plasma cell neoplasms had worse outcomes. The mortality was 37% among the 106 patients with plasma cell neoplasm. A similar overall COVID-19 mortality rate of 34% was also observed in a large retrospective International Myeloma Society Study on 650 patients with multiple myeloma (MM) from 10 different countries [4]. Multivariate analysis showed that age, high-risk disease by FISH, renal disease and suboptimal control of myeloma were independent predictors of worse outcome. Lower mortality rates were observed in patients with myeloproliferative neoplasms (MPN) (myelofibrosis, polycythemia vera, essential thrombocytosis). In a large European observational retrospective study, 50 of the 175 patients (29%) with MPN died at a median of 9 days after the diagnosis of COVID-19 [5]. Mortality was higher than in the general population and reached 48% in patients with myelofibrosis. There are also clinical and laboratory observations on a protective role of tyrosine kinase inhibitors (TKIs) versus SARS-CoV-2 infection in patients with chronic myeloid leukemia (CML) [6, 7].

COVID-19 vaccines recommendations in hematological patients have been provided by several scientific societies [8–14]. In summary, mRNA vaccines should be preferred, and while there are no particular expected problems in terms of safety, the efficacy data have to be verified in light of potentially reduced immunogenicity in immunocompromised patients. These patients are on the one hand at higher risk of COVID-19 mortality, on the other hand at higher risk of ineffectiveness from vaccination, both aspects due to deep humoral and cell-mediated immunosuppression related to treatments and underlying disease. A real uncertainty exists about the optimal timing, dosing and capacity of some subgroups of hematological patients to efficiently respond to the COVID-19 vaccination [15, 16]. Several guidelines in pre-COVID-19 era recommended that vaccines are administrated at least 3–6 months after the end of an anti-lymphoid therapy in order to avoid their likely futility [17, 18]. Two consensus-based recommendations in MM patients were very recently published by the European Myeloma Network and an experts Italian panel [19, 20]. Both publications highlighted that the response in myeloma patients is often less vigorous.

On the basis of the Italian national plan against COVID-19, a RNA vaccine should be offered as soon as possible to onco-hematological patients in treatment with immunosuppressive or myelosuppressive drugs or within 6 months from the end of such treatment, and to stem cell transplanted patients after 3 months from transplant [21].

According to this plan, since March 2021 BNT162b2 mRNA vaccination (Pfizer-BioNTech) has started at our institute for patients with hematological malignancies on active treatment.

Herein we report preliminary data on 42 patients with MM and 50 with myeloproliferative malignancies (MPM) (Philadelphia-negative MPN *n* = 30 and CML *n* = 20), who were vaccinated and evaluated for anti-SARS-CoV-2-neutralizing IgG titer on day of first injection, on day of second injection (i.e., after 3 weeks) and after 2 weeks from second injection, according to a monocentric institutional study. Serological responses were compared with those observed in 36 elderly subjects aged over eighty not suffering from cancer.

## Methods

### Study design, objectives and subjects

In order to evaluate the immunogenicity of BNT162b2 vaccine in different populations, a monocentric prospective cohort study was formally approved by the IRCCS Central Ethical Committee of Regione Lazio in January 2021 (Prot. N-1463/21). The study aims to measure antibody titers, seroconversion rates and trends in patients with solid cancers and hematological malignancies, elderly subjects over 80 aged and healthy health workers. Neutralizing IgG titers anti-SARS-CoV-2 was evaluated at basal (day 0, first injection, time point [TP]0), after 3 weeks (day 21, second injection, TP1) and two weeks post-booster (day 35, TP2). Subsequently, titration TP is expected at 12 (TP3), 24 (TP4) and 52 (TP5) weeks from the first injection. Data on safety were also collected on TP1 and TP2 by interview. Local and systemic side effects were registered. Symptoms and signs were graded as follows: none, mild (no interference with daily activities), moderate (interference with normal activity), severe (impediment of normal activities or need to medical advice). All participants were asked to provide nose and throat swabs on each defined TP and in a small sample also peripheral blood mononuclear cells for subsequent studies of T-cell response. The study was conducted in compliance with Helsinki Declaration and Good Clinical Practice, and all subjects signed a specific written informed consent before study enrollment. We decided to focus the analysis initially on those patients with MM and MPM (i.e., CML and Philadelphia-negative MPN) who had received the first vaccine injection on March 2 or 9 or 16 or 24, 2021 (half-day weekly slots were dedicated to hematological patients) and for whom all tests’ results at each of the first four TPs were available. Patients with a positive basal IgG titer were excluded from the analysis. A comparison with a sample of elderly subjects over 80 aged not suffering from cancer was foreseen. This latter population had the function of representing the control arm, given the median age close to the median age of the two hematological cohorts.

### Schedule of vaccination and treatments

The schedule of vaccination was classically based on two intramuscular injections of 30 µg per dose of BNT162b2 vaccine three weeks apart. Neither suspension nor dose modification of the therapy schemes was planned in any treatment set.

### Serological test and definition

LIAISON® SARS-CoV-2 S1/S2 IgG by DiaSorin®, Saluggia, Italy.

The LIAISON® SARS-CoV-2 S1/S2 IgG test is a quantitative chemiluminescent immunoassay (CLIA), fully automated on LIAISON® XL platform, for the detection of IgG antibodies against the subunits S1 and S2 of SARS-CoV-2 spike protein. The subunits S1 and S2 are responsible for binding and fusion of virus to host cell, respectively, and are both targets of neutralizing antibodies.

According to the manufacturers’ technical manual, the result of a LIAISON® SARS-CoV-2 S1/S2 IgG test has to be reported as positive with a signal of 15 AU/mL or higher, equivocal between 12 and 15 AU/mL and negative < 12 AU/mL [22]. The cutoff to discriminate the positives was individuated on the basis of a level of concordance of 94.4% between the LIAISON® IgG titer of 15 AU/mL and the Plaque Reduction Neutralization Test 90% (PRNT90) at 1:40 ratio. To date, PRNT90 is a gold standard for evaluating the relative concentrations of virus-specific neutralizing antibodies. At a LIAISON® value of 80 AU/mL a concordance of 100% with a 1:160 PRNT90 titer was observed. The robustness of the evidences of concordance between this CLIA and the PRNT90 allowed to LIAISON® SARS-CoV-2 S1/S2 IgG by DiaSorin® to be approved by FDA on April 2020. Clinical and analytic performance of this automated serological test identifying SARS-CoV-2 S1/S2-neutralizing IgG in a semi-quantitative manner was published on September 2020 [23].

For the analysis’ purposes, the cutoff of 15 AU/mL indicated by DiaSorin® was considered valid to discriminate responders from non-responders to vaccination. In light of this, the terms serological response (i.e., positive test with a signal of 15 AU/mL or higher), serological conversion (i.e., from a titer below to a titer above the cutoff of 15 AU/mL) and serological protection (i.e., by identification of neutralizing IgG as proved by LIAISON® SARS-CoV-2 S1/S2 IgG test) have to be considered equivalent in our report.

All blood samples were centrifuged at 3000 relative centrifuge force (RCF) for 10 min and sera analyzed within 4 h of centrifugation.

### Molecular testing

Bosphore® Novel Coronavirus (2019-nCoV) Detection Kit v3, Anatolia geneworks®, Istanbul, Turkey.

Bosphore® Novel Coronavirus (2019-nCoV) Detection Kit v3 detects and characterizes 2019-nCoV in human respiratory samples. Fluorescence detection is accomplished using FAM, HEX, Texas RED and Cy5 filters. SARS-CoV-2 is detected by three regions of the virus in a single reaction: E gene is used for screening purpose, where SARS-CoV-2 and also the closely related coronaviruses are detected, and the orf1ab target region and N gene region are used to discriminate SARS-CoV-2 specifically. Before amplification a fast extraction is performed, which does not require a separate extraction but only a pre-treatment that takes less than 10 min.

Real-time PCR was performed with Montania 4896® thermal cycler.

Swabs were tested within 4 h from collection.

### Statistical analysis

Cohorts were compared using Fisher’ exact test for categorical data and the Mann–Whitney test for continuous variables. To evaluate the effect of the second dose of vaccine in each cohort, Wilcoxon test was used for comparing geometric mean concentration (GMC) and geometric mean ratio between TP1 and TP2 and McNemar test for comparing response rate between TP1 and TP2. A generalized multivariate linear model (GLM) was implemented to evaluate the correlation between logarithm of IgG titer at TP2 and gender, age and cohorts. Odds ratio of significant variables for response to vaccine was assessed. Multivariate analysis using a logistic regression model was planned in case of multiple variables significant for response at univariate analysis. Confidence intervals for GMC were calculated based on logarithm of titers. All P values were two-sided, and those ≤ 0.05 were considered statistically significant. Analysis was performed using the software package SPSS (version 20).

## Results

### Enrollment

**Fig. 1:**
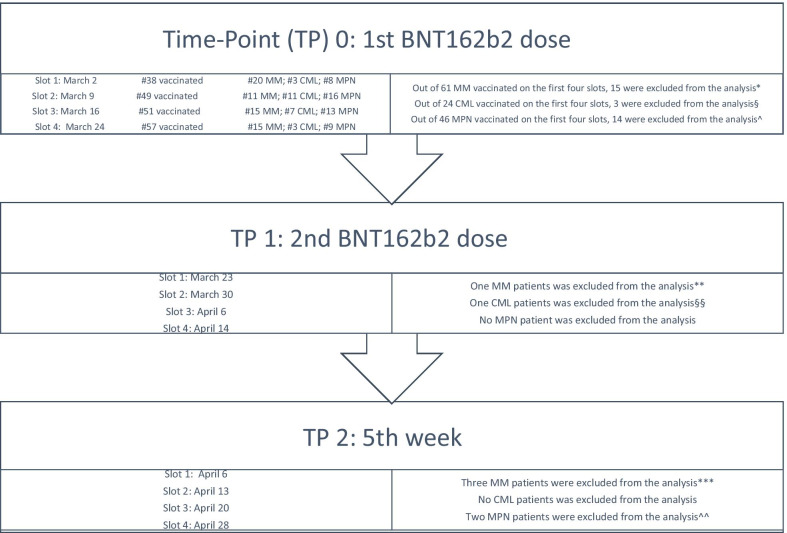
Participants flow diagram. *Fifteen MM patients were excluded from analysis at TP0 due to refusal of the study (*n* = 12) or no systemic ongoing therapy (*n* = 3; one patient on radiotherapy and two on follow-up post-VMP). **One MM patient did not receive the second dose due to concomitant bacterial infection. ***Three MM patients did not perform titration at TP2. §Three CML patients were excluded from analysis at TP0 due to refusal of the study (*n* = 2) or anti-SARS-CoV-2 IgG positivity at basal (*n* = 1). §§One CML patient did not receive the second dose because lost to follow-up. ^Fourteen MPN patients were excluded from analysis at TP0 due to refusal of the study (*n* = 12), no systemic ongoing therapy (*n* = 1) or anti-SARS-CoV-2 IgG positivity at basal (*n* = 1). ^^Two MPN patients did not perform titration at TP2

Out of 360 hematological patients who had to be vaccinated as a priority according to national plan, 78 were affected by MM, and 106 by MPM. The analysis was carried out on 42 MM and 50 MPM patients. These 92 patients were those who, on first four vaccination slots (first dose on March 2, 9, 16, 24), had signed informed consent, had not a positive test at basal and for whom all tests’ reports were available. The participants flow diagram is shown in Fig. 1.

The control cohort was represented by 36 elderly subjects over 80 aged not suffering from cancer who had completed sampling on TP2.

### Characteristics of the study cohorts

Table 1 shows the demographic and hematological characteristics of the study’ cohorts. The age was similar between MM and MPM patients, with a median of 73 and 70 years, respectively, lower than that in control population over 80 aged by definition. Gender distribution was equivalent among the cohorts. Body mass index (BMI) was similar between MM and MPM patients. Lines of therapy, lymphocyte and neutrophils counts at basal, interval between the beginning of the ongoing treatments and vaccination were significantly different between the groups as expected, being intrinsically related to the primitive involvement of different hematopoietic lineages and to different therapeutic approaches.

**Table 1.** Demographic and hematological characteristics of the study’ cohorts.

Characteristics	Cohorts			*P*
	Elderly control (*n *= 36)	Multiple myeloma (*n *= 42)	Myeloproliferative malignancy (*n *= 50)	
Age, median (range)	81 (79–87)	73 (47–78)	70 (28–80)	**<** 0.001*
Age, median (range)		73 (47–78)	70 (28–80)	0.056
Sex, F/M	18/18	19/23	24/26	0.914
BMI (kg/sm^2^), median (range)	Not available	24.3 (19.4–36.1)	24.8 (18.6–39.7)	0.872
Time (months) from diagnosis to vaccination, median (range)		72 (3–213)	74 (2–306)	0.266
Time (months) from beginning of ongoing therapy to vaccination, median (range)		9 (1–111)	39 (1–209)	**<** **0.001**
Number of lymphocytes/µL, median (range)		1150 (430–2300)	1810 (240–4300)	**<** **0.001**
Number of neutrophils/µL, median (range)		1800 (700–23100)	3580 (1400–34400)	**<** **0.001**
Lines of therapy, median (range)		2 (1–5)	1 (1–4)	**0.003**
Ongoing treatments		PI-based *n *= 9 VTD *n *= 2 VMP *n *= 2 VCD *n *= 1 IRD *n *= 2 KRD *n *= 1 PVD *n *= 1	Hydroxycarbamide *n *= 20	
		Daratumumab-based DRD *n *= 14	Tyrosine kinase inhibitors *n *= 20 Imatinib *n *= 7; nilotinib *n *= 7; dasatinib *n *= 4; bosutinib *n *= 2	
		Imids-based *n *= 19 Lenalidomide+dex *n *= 17 Pomalidomide+dex *n *= 1 ERD *n *= 1	Ruxolitinib *n *= 6	
			Interferon alpha *n *= 2	
			Anagrelide *n *= 2	
Diagnosis			Chronic myeloid leukemia *n *= 20	
			Essential thrombocythemia *n *= 11	
			Myelofibrosis *n *= 8	
			Polycythemia vera *n *= 11	

BMI: body mass index; sm^2^: square meter^2^; PI: proteasome inhibitor; VTD: bortezomib thalidomide dexamethasone; VMP: bortezomib melphalan prednisone; VCD: bortezomib cyclophosphamide dexamethasone; IRD: ixazomib lenalidomide dexamethasone; KRD: carfilzomib, lenalidomide, dexamethasone; PVD: bortezomib, pomalidomide, dexamethasone; DRD: daratumumab lenalidomide dexamethasone; ERD: elotuzumab lenalidomide dexamethasone; Imids: immunomodulatory imide drugs.

*Kruskal–Wallis test

### Comparison of serological response to BNT162b2 vaccination on TP1 and TP2 among the cohorts

In Table 2 is reported the GMC of IgG on TP0, TP1 and TP2 and seroconversion rate on TP1 and TP2 in MM and MPM cohorts in comparison with the elderly control cohort. Whereas on TP1 no statistical difference was observed between MPM patients and control cohort, MM patients responded significantly less than the elderly control subjects. Namely, antibody GMC in elderly controls was 17.1 AU/mL versus 7.5 in MM (*p* < 0.001) and 16.2 in MPM patients (*p* = 0.837), while seroconversion rate was 19/36 (52.8%) in controls compared to 9/42 (21.4%) in MM (*p* = 0.005) and 26/50 (52.0%) in MPM patients (*p* = 1). On TP2 the titers increased at a different extent in patients with MM and MPM compared to elderly control population. Namely, antibody GMC in elderly controls was 353.3 AU/mL versus 106.7 in MM (*p* = 0.003) and 172.9 in MPM patients (*p* = 0.049), while seroconversion rate was 36/36 (100%) in controls compared to 33/42 (78.6%) in MM (*p* = 0.003) and 44/50 (88.0%) in MPM patients (*p* = 0.038). A further indicator, useful to demonstrate the relative increase of IgG titer after the booster in each cohort, is represented by the geometric mean ratio, i.e., the ratio between GMC on TP2 and TP1, calculated by TP2-GMC/TP1-GMC. In comparison with a ratio of 19.7 for elderly controls, the ratio was 13.1 for MM patients (*p* = 0.288) and 10.0 for MPM patients (*p* = 0.015). The impact of gender, age and cohort on the outcome at TP2 in terms of logarithm of IgG titer was evaluated through a GLM. No gender effect was observed (*p* = 0.913), while there was a significant trend to lower response according to increase in age (*p* < 0.001) and for disease cohorts with respect to elderly subjects (MM: *p* < 0.001 and MPM: *p* < 0.001).

**Table 2:** Antibody response by GMCs, geometric mean ratios and response rates in multiple myeloma and myeloproliferative malignancies in comparison with elderly controls.

	Multiple myeloma (*n *= 42)	Myeloproliferative malignancy (*n *= 50)	Elderly controls (*n *= 36)
TP0 day 0 (1st dose)			
GMC (95% CI), AU/mL	4.2 (3.9–4.6)	4.6 (4.2–5.2)	3.8 (3.8–3.8)
TP1 day 21 (2nd dose)			
GMC (95% CI), AU/mL [*p*]	7.5 (5.6–10.4) [*p* < 0.001]	16.2 (11.7–22.3) [*p *= 0.837]	17.1 (12.0–24.1)
Seroconversion rate (i.e., responders), *n* (%) [*p*]	9 (21.4) [*p *= 0.005]	26 (52.0) [*p *= 1]	19 (52.8)
TP2 day 35			
GMC (95% CI), AU/mL [*p*]	106.7 (62.3–179.7) [*p *= 0.003]	172.9 (106.5–257.0) [*p *= 0.049]	353.3 (255.6–470.0)
GM ratio (TP2-GMC/TP1-GMC) (95% CI) [*p*]	13.1 (8.2–21.1) [*p *= 0.288]	10.0 (7.1–13.4) [*p *= 0.015]	19.7 (13.5–28.4)
Seroconversion rate (i.e., responders), *n* (%) [*p*]	33 (78.6) [*p *= 0.003]	44 (88.0) [*p *= 0.038]	36 (100)

GMC, geometric mean concentration (95% confidence interval); TP, time point.

### Comparison of serological response to BNT162b2 vaccination on TP1 and TP2 within each cohort

**Fig. 2:**
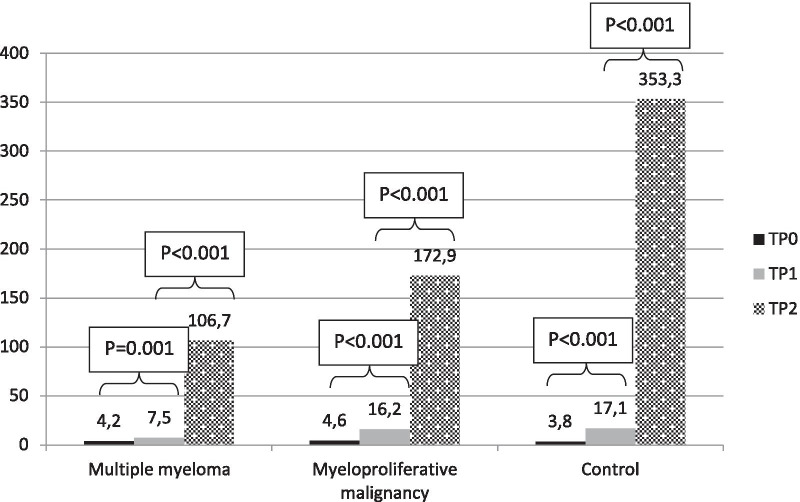
IgG geometric mean concentrations on TP0, TP1 and TP2 in each cohort, AU/mL. TP0 is day of 1st dose, TP1 is day of 2nd dose (3rd week after 1st dose), TP3 is 5th week after 1st dose

**Fig. 3:**
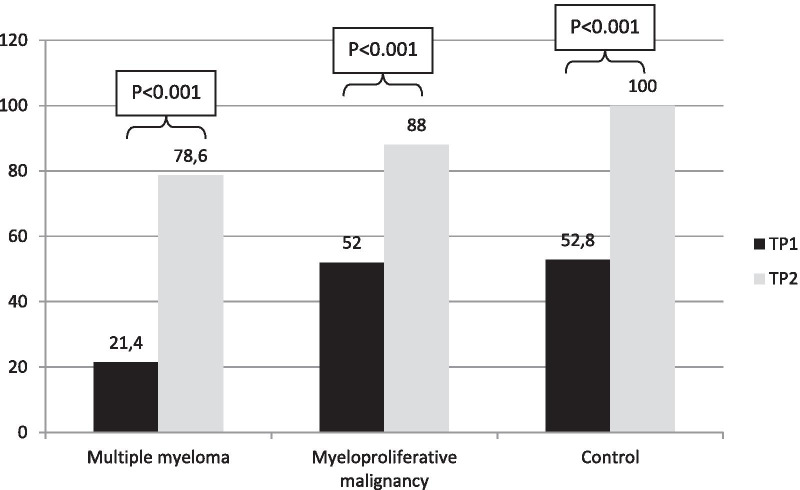
Response rates at cutoff of 15 AU/mL on TP1 and TP2 in each cohort, %. TP1 is day of 2nd dose (3rd week after 1st dose), TP3 is 5th week after 1st dose

Figures 2 and 3 show the GMC of IgG and the response rate at cutoff of 15 AU/mL on TP1 and TP2 within each cohort, respectively. An increment of both IgG GMCs and proportions of responders was elicited in all the cohorts by the booster. In MM patients there was a significant increment of both IgG GMC (from 7.5 AU/mL to 106.7; *p* < 0.001) and proportion of responders (from 21.4 to 78.6%; *p* < 0.001). In MPM patients, GMC of IgG increased significantly after the booster (from 16.2 AU/mL to 172.9; *p* < 0.001), as the proportion of responders (from 52 to 88%; *p* < 0.001). A more pronounced trend was observed in the increase of IgG GMC (from 17.1 AU/mL to 353.3; *p* < 0.001) and response rate (from 52.8 to 100%; *p* < 0.001) in elderly subjects.

**Fig. 4:**
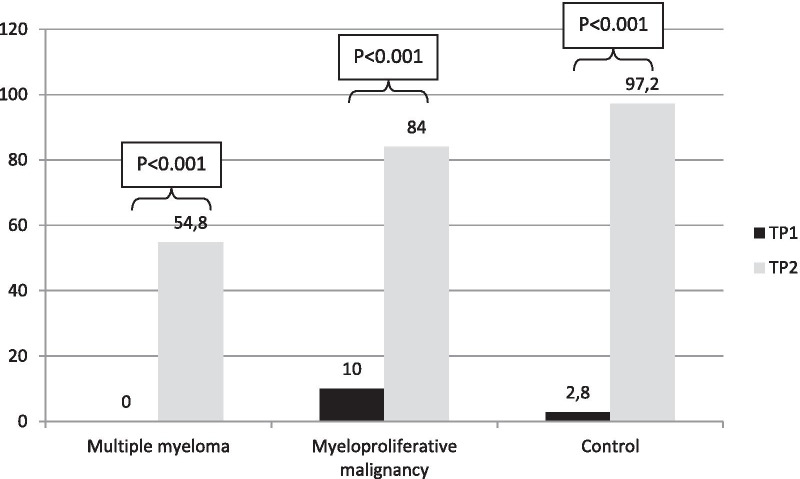
Response rates at hypothetical cutoff of 80 AU/mL on TP1 and TP2 in each cohort, %. TP1 is day of 2nd dose (3rd week after 1st dose), TP3 is 5th week after 1st dose

Figure 4 shows what the response rate would be using a cutoff of 80 AU/mL instead of 15 on TP1 and TP2 within each cohort. On TP2, the proportion of responders in MM patients would fall to 54.8%, and in MPM patients and elderly controls it would remain almost unchanged (84% instead of 88% and 97.2% instead of 100%, respectively).

### Predictors of serological response to BNT162b2 vaccine within the patient cohorts

Next step was to analyze individually the two patient cohorts in order to identify possible predictors of response.

On TP2, in the cohort of patients with CML and MPN, only 6 patients (four with myelofibrosis of which three on ruxolitinib, one with essential thrombocythemia and one with polycythemia vera) did not respond to vaccine and did not seroconvert above the cutoff of 15 AU/mL. No analysis on variables associated with response to vaccine was done due to limited number of events (i.e., no response).

In patients with MM, on TP2, in univariate analysis the likelihood of response was significantly associated only with the type of treatment (Table 3). Patients on active treatment with proteasome inhibitors-based and imids-based therapies, alone or in combo, without daratumumab, had a likelihood of response higher compared to those on daratumumab (92.9% vs 50%; *p* = 0.003).

**Table 3:** Predictors of response to vaccine on TP2 in multiple myeloma patients.

Variables	Univariate analysis	
	Responders	*p*
Age		0.269
> 73 years (*n *= 20)	14 (70%)	
< 73 years (*n *= 22)	19 (86.4%)	
Gender		1
M (*n *= 23)	18 (78.3%)	
F (*n *= 19)	15 (78.9%)	
BMI		0.130
> 24.3 (*n *= 21)	19 (90.5%)	
< 24.3 (*n *= 21)	14 (66.7%)	
Lines of therapy		0.271
1 (*n *= 17)	15 (88.2%)	
> 1 (*n *= 25)	18 (72%)	
Lymphocyte count		0.454
> 1150/µL (*n *= 21)	18 (85.7%)	
< 1150/µL (*n *= 21)	15 (71.4%)	
Time from diagnosis to vaccination		1
> 72 months (*n *= 21)	17 (81%)	
< 72 months (*n *= 21)	16 (76.2%)	
Time from the start of ongoing therapy to vaccination		1
> 9 months (*n *= 20)	16 (80%)	
< 9 months (*n *= 22)	17 (77.3%)	
Treatment		**0.003**
Daratumumab-based (*n *= 14)	7 (50%)	
PI-based/Imids-based alone or in combo without daratumumab (*n *= 28)	26 (92.9%)	

CI, confidence interval; BMI, body mass index; PI, proteasome inhibitors; Imids, immunomodulatory imide drugs; TP, time point.

### BNT162b2 vaccine safety

Side effects after first and second dose are reported in Table 4. Mild pain at the injection site within few days was the most common side effect in all the cohorts. Only two patients reported severe pain after 2nd dose, and no serious adverse event was registered.

**Table 4.** Side effects in pooled cohorts

	After 1st dose		After 2nd dose		
	Mild	Moderate	Mild	Moderate	Severe
Local					
Pain, %	20		13	3	2
Tenderness, %	10		7	1	
Systemic					
Fever, %			3	1	
Headache, %	1		2		
Malaise, %	3	2	1		
Myalgia, %	1			1	
Chills, %			1	1	

### SARS-CoV-2 infection during the study period

No enrolled patient resulted positive at nose and throat swabs which were collected on each TP.

## Discussion

Two mRNA vaccines have been approved by FDA and EMA, having demonstrated to enhance effectively immunogenicity against SARS-CoV-2 in general population [24, 25]. In particular, BNT162b2 was 95% effective in preventing COVID-19 [25]. Hematological patients were not enrolled in the registration trials. The extent of a hypothetical reduced efficacy of mRNA vaccines to elicit an immunological response respect to general population, although expected in these immunocompromised patients, is widely unknown.

Four issues regarding vaccination in hematological patients become dramatically crucial in a pandemic like that by COVID-19.

Firstly, defining the positivity cutoff of reliable tests that measure neutralizing antibodies is the methodological premise for any subsequent reasoning. LIAISON® SARS-CoV-2 S1/S2 IgG test by DiaSorin® was validated as a marketable CLIA by FDA. The positivity cutoff for neutralizing IgG was defined using data from wild infection in COVID-19 individuals. Are there consistent immune-biological or technical reasons why it might not be valid even after vaccination or in immunocompromised patients? An antibody titer evaluated with LIAISON® SARS-CoV-2 S1/S2 IgG above 15 AU/mL has demonstrated a concordance level with a gold standard for the evaluation of the virus-specific antibody neutralizing power represented by PRNT90 at 1:40 ratio of 94.4%, while above 80 AU/mL the level of agreement with 1:160 PRNT90 titer was 100%. There are no valid reasons in our opinion to doubt that this commercial CLIA is reliable in detecting the neutralizing antibody concentration at the cutoff of 15 AU/mL even after vaccination and in immunocompromised patients.

Secondly, defining the optimal timing of vaccination is a paramount challenge. There is no doubt that vaccinating patients at least one months before starting treatment or 3–6 months after the end of therapies would be optimal. Unfortunately, there are cancer patients who cannot stop ongoing therapies or who cannot wait. There is a need to take responsibility what to do for such vulnerable patients, particularly those receiving lympho-depleting treatments. Our policy was to vaccinate all patients in active treatment according to indication of the national plan as soon as possible, without substantial modifications or delay in the administration of the ongoing treatment scheme. Therefore, it should be noted that all the data from our study were obtained in patients on active treatment for their hematological neoplasm.

Thirdly, in patients with hematological malignancies immune deficiencies are heterogeneous. Patients with lymphoproliferative disorders and those with myeloid neoplasms suffer from distinct immune deficiencies and receive different treatments that variously affect the vaccine response and that are notably different compared with the past. Our choice was to focus on two different set of diagnoses, poles apart in many ways from each other.

Fourthly, although the incidence of proven SARS-CoV-2 infection in the vaccinated subjects remains the preferred clinical efficacy endpoint of a vaccine, the seroprotection rate has to be regarded as an acceptable surrogate endpoint in immunocompromised populations. Therefore, it was important to immediately verify the immunogenicity of a mRNA vaccine in hematological patients and to compare the data with those obtained from a non-neoplastic elderly control population similar aged.

At the best of our knowledge, this is the first report focusing on the humoral response to mRNA BNT162b2 vaccination in patients with MM and MPM.

As proof of concept, the mRNA BNT162b2 vaccine has demonstrated to be immunogenic in immunocompromised patients with MM and MPM on active treatment, eliciting a significant serological response. The GMCs of IgG anti-S1/S2 subunits of SARS-CoV-2 spike protein significantly rose after the booster in all the cohorts, at a significantly different extent among the cohorts. In fact, the highest absolute increase of IgG after the booster, expressed in terms of GMC, was reached in the elderly controls, the lowest in the MM patients, with MPM patients in the middle.

MPM patients responded to vaccine reaching proportions of seroconversion of 88%, significantly lower compared to controls over 80 aged (100%; *p* = 0.038). MM patients responded partially, with a seroconversion rate of 78.6% after the booster significantly inferior to elderly controls (*p* = 0.003). Therefore, a near-complete seroprotection was achieved in MPM patients, whereas the booster worked at minor extent in patients with MM. Furthermore, such seroprotection in MM patients does not appear robust, since almost one-third of MM patients, defined responders at the cutoff of 15 AU/mL, would be defined not responders by raising the cutoff at 80; in fact, at such cutoff, seroprotection rate decreases from 78.6 to 54.8%, contrary to what happens in the other two cohorts.

These observations, although exciting, are not surprising.

MM is one of the most immunologically defective conditions among cancers per se. A recent review summarized dysfunctions of immune cells in the myeloma niche [26]. From cross-talk between tumor plasma cells and bone marrow (BM) microenvironment derives an immunosuppressive context characterized by loss of effective antigen presentation, dysfunction of effector cells, expansion of immunosuppressive cells. As a consequence, the immunogenic effectiveness of vaccines in MM is a hot issue. In patients with MM, a yearly single dose of inactivated influenza vaccine is recommended by ECIL 7 guidelines in light of controversial benefit of a booster, and variable seroconversion rates, mostly between 20 and 25%, have been observed [27]. However, at least in two studies dedicated to MM patients, which measured the rates of seroprotection after each of two doses of influenza vaccine, the rates of patients with protective titers rose significantly post-booster, reaching proportion around 70% [28, 29]. Active disease requiring therapy was associated with lower likelihood for a serological response. Also in a randomized placebo-controlled trial of adjuvanted recombinant zoster vaccine (two doses 1–2 months apart) administered during or after immunosuppressive cancer treatment in 569 patients with hematological malignancies, including 132 MM patients, the humoral response at month 2 was 80%. Unfortunately, separate results for MM patients were not reported [30]. These percentages are not different from that observed in our study. It should be extremely relevant to establish whether there are identifiable biomarkers predicting the vaccine response. From this point of view, treatment was significantly associated with response to BNT162b2 vaccine. Patients on active treatment with proteasome inhibitors-based and imids-based therapies, alone or in combo, without daratumumab, had a significant likelihood of responding to vaccine (92.9%). On the contrary, daratumumab in combo with lenalidomide was significantly associated with a lower response rate (50%). This is in line with the current evidences. In fact, even if treatments on the one hand restore a condition of partial immune-competence by direct anti-neoplastic effects and disruption of interaction between plasma cells and BM microenvironment, on the other hand, selectively impacts on many cellular actors of the immune system [26]. At one end of the spectrum, imids promote immune activation by functional enhancement of T and NK cells, increase of Th1 cytokine production, reduction of Treg activity, improvement of dendritic cells maturation and functions, enhancement of antibody-dependent cell-mediated cytotoxity [31–33]. At the other end, daratumumab (and isatuximab) targets CD38 on the population of normal and tumor plasma cells, in this way reducing vaccines immunogenicity by direct depletion of antibody producer cells. However, a recent paper reported that IgG levels and induction of protective antibody titers were intact against *Streptococcus Pneumoniae*, *Hemophilus Influenzae B* and seasonal influenza at a median of two months after treatment, probably by escape from daratumumab of a subset of plasma cells due to a reduced expression of CD38 [34]. The policy of not modifying the therapy scheme before, during and after the vaccine administration suggests cautiousness in the interpretation of our data, which might be even better outside an active therapy setting. However, the fact that not all MM patients responded to vaccine, and that those who responded did it not robustly, poses great concerns and turns to the opportunities offered by the new mRNA vaccination platforms. For example, repeated booster of mRNA vaccine, at intervals to be defined, could theoretically reinforce the humoral response. Clinical trials should determine the optimal timing and dosing schedule of BNT162b2 vaccination in this patient group. Presently, it appears clear that increasing measures are urgently required to consolidate the scarce immunological protection achieved by vaccination in these MM patients. For the purposes of a correct risk management, it appears important to highlight both the need to vaccinate the family context, and the need that no misleading message should be conveyed through the mass media on the complete effectiveness to prevent COVID-19 by vaccination of such population, who still has to be protected by virtuous behaviors based on the use of social distancing, face mask wearing and hand hygiene.

The low percentages of SARS-CoV-2 infection and severe COVID-19 which were observed in patients with CML and chemo-free-treated Philadelphia-positive acute lymphoblastic leukemia might be related to a protective action exerted by TKIs [6, 35]. It has been recently reported that genes with anti-infective effect (for example CCL5, CD28, IFN gamma) are upregulated and others with pro-infective effect (for example ARC-1, FUT4) are downregulated by TKIs in patients with CML [7]. The expression profiles of these genes involved in inflammation and immunity were measured after six months of treatment with imatinib, and the results supported the idea of an absence of immunological damage after a median-length treatment with TKIs. From our data, all the 20 patients with CML on TKIs responded robustly to BNT162b2. In Philadelphia-negative MPN patients, the COVID-19 mortality was higher than in general population, particularly in myelofibrosis, and was correlated with the interruption of ruxolitinib, probably due to enhancement of cytokine release syndrome which in turn may lead to multiorgan failure [5]. No conclusion on how ruxolitinib works on vaccine immunogenicity may be drawn from our population because only six patients were on ruxolitinib at the time of vaccination. Also the number of patients with myelofibrosis (*n* = 8) was too small to be analyzed as variable in its own right; however, 4 out of these 8 patients did not respond to vaccine. Overall, data from other vaccination contexts in patients with CML or Philadelphia-negative MPN are limited to inactivated influenza vaccine and pneumococcal polysaccharide 23-valent vaccine and to patients not receiving ruxolitinib [27]. In particular, in patients with CML, a strategy based on two doses of H1N1 vaccine administered three weeks apart allowed to increase the seroprotection rate from 85 to 95%, proportions not so different by those observed after vaccination with BNT162b2 in our cohort of 20 CML patients [36].

Our study has some limitations: First of all, the limited number of patients impacts on the statistical power when we look at some variables as predictors of response, although some trends seem clear (i.e., myelofibrosis as variable potentially associated with a lower likelihood of response). Moreover, the limited observation period and the absence of concomitant investigations on the T-cell response do not allow to draw firm conclusions on two paramount questions which remain unanswered, namely whether responding patients actually develop true protection from COVID-19 and how long this protection extends over time. Combining cellular and humoral measures of vaccine efficacy may increase the ability to predict the risk of COVID-19. Evidence exists of robust memory T-cell responses in patients with MPN following SARS-CoV-2 infection [37]. The extent and robustness of such T-cell response after vaccination with BNT162b2 is a crucial point to verify in both MM and MPM patients. Recently, delayed and impaired B- and T-memory cells responses were reported at three weeks and three months after a single dose of seasonal influenza A vaccine in MPN compared to healthy controls [38].

## Conclusions

At five weeks, the population with MPM responded robustly and near completely to BNT162b2 vaccine with the exception of patients with myelofibrosis, closing the gap from elderly controls. In MM patients, who responded partially and less robustly, anti-CD38-based therapy impacted negatively on the vaccination. The new mRNA platforms allow to hypothesize different administration schedules based on closer and/or personalized booster, opening the doors also in this scenario to a precision medicine. In light of the scarce immunological protection by vaccine, in these MM patients on active treatment with anti-CD38 monoclonal antibodies, IgG titer monitoring should be implemented and social distancing and mask wearing maintained regardless of vaccination status. Primary prophylaxis by anti-SARS-CoV-2 monoclonal antibodies should be explored in trials for MM patients at higher risk of no response. However, confirmatory and more exhaustive studies are urgently needed.

## Data Availability

The datasets used and/or analyzed during the current study are available from the corresponding author on reasonable request.

## Abbreviations

BM: Bone marrow
BMI: Body mass index
CML: Chronic myeloid leukemia
GLM: Generalized multivariate linear model
GMC: Geometric mean concentration
MM: Multiple myeloma
MPM: Myeloproliferative malignancies
MPN: Myeloproliferative neoplasms (Ph negatives)
PRNT90: Plaque Reduction Neutralization Test 90%
RCF: Relative centrifuge force
